# Supplementary material for: Spatiotemporal patterning of photoresponsive DNA-based hydrogels to tune local cell responses
Source: Nat Commun. 2021 Apr 22;12:2364. doi: 10.1038/s41467-021-22645-8 (PMC8062675; doi:10.1038/s41467-021-22645-8)
Supplement: Supplementary file 1 — Supplementary Information [file 41467_2021_22645_MOESM1_ESM.pdf]

# Supporting Information

Spatiotemporal patterning of photoresponsive DNA-based hydrogels to tune local cell responses

Fujian Huang[1], Mengxi Chen[1], Zhixin Zhou[2], Ruilin Duan[1], Fan Xia[1] and Itamar Willner[2]

[1] State Key Laboratory of Biogeology and Environmental Geology, Engineering Research Center of Nano-Geomaterials of Ministry of Education, Faculty of Materials Science and Chemistry, China University of Geosciences, Wuhan 430074, China
[2] Institute of Chemistry, Center for Nanoscience and Nanotechnology, The Hebrew University of Jerusalem, Jerusalem 91904, Israel

Correspondence should be addressed to F.H. (email: huangfj@cug.edu.cn), F.X. (email: xiafan@hust.edu.cn) and I.W. (email: itamar.willner@mail.huji.ac.il).

## 1. Supplementary Tables

**Supplementary Table 1.** Self-complementary DNA sequences (photocleavable or not) used as crosslinker in hydrogel formation process.

| **Name** | **Sequence (5'→3')** |
|---|---|
| **1** | **Acrydite**-AATGCTCGCGCGAGCA |
| **4** | **Acrydite**-AA//**PC**//TGCTCGCGCGAGCA |

**Supplementary Table 2.** Photocleavable single-strand DNA and FAM modified complementary strand.

| **Name** | **Sequence (5'→3')** |
|---|---|
| **2** | **Acrydite**-TTTTT//**PC**//GGCTATAGCACATGGGTAAAACGAC |
| **2'** | **FAM**-GTCGTTTTACCCATGTGCTATAGCC |

**Supplementary Table 3.** Photoactivatable hairpin structured DNA, TAMRA modified invading DNA strand and TAMRA modified invading DNA strand with MUC-1 aptamer sequence.

| **Name** | **Sequence (5'→3')** |
|---|---|
| **3** | **Acrydite**-TTTTCCGGAATTCCGCTTCTAGTT//**PC**//GCGGAATTCCGGAAAA |
| **3'** | **TAMRA**-AACTAGAAGCGGAATTCCGGAAAA |
| **3''** | **TAMRA**- <u>GCAGTTGATCCTTTGGATACCCTGG</u>TTTTTTTTTAACTAGAAGCG GAATTCCGGAAAA |

**Note:** Underlined is the sequence of MUC-1 aptamer.

**Supplementary Table 4.** DNA sequences used in the site-specific swelling process of patterned hydrogels for hydrogel pattern regulation.

| **Name** | **Sequence (5'→3')** |
|---|---|
| **5** | **Acrydite**-TAAGTTCGCTGTGGCACCTGCACG |
| **5'** | **Acrydite**-GTACAA//**PC**//CGTGCAGGTGCCACAGCGTGGATC |
| **H1** | GATCCGCGCTGTGGCACCTGCACGCACCCACGTGCAGGTGCCACAGCGAA CTTA |
| **H2** | TGGGTGCGTGCAGGTGCCACAGCGTAAGTTCGCTGTGGCACCTGCACGTTG TAC |

## 2. Supplementary Figures

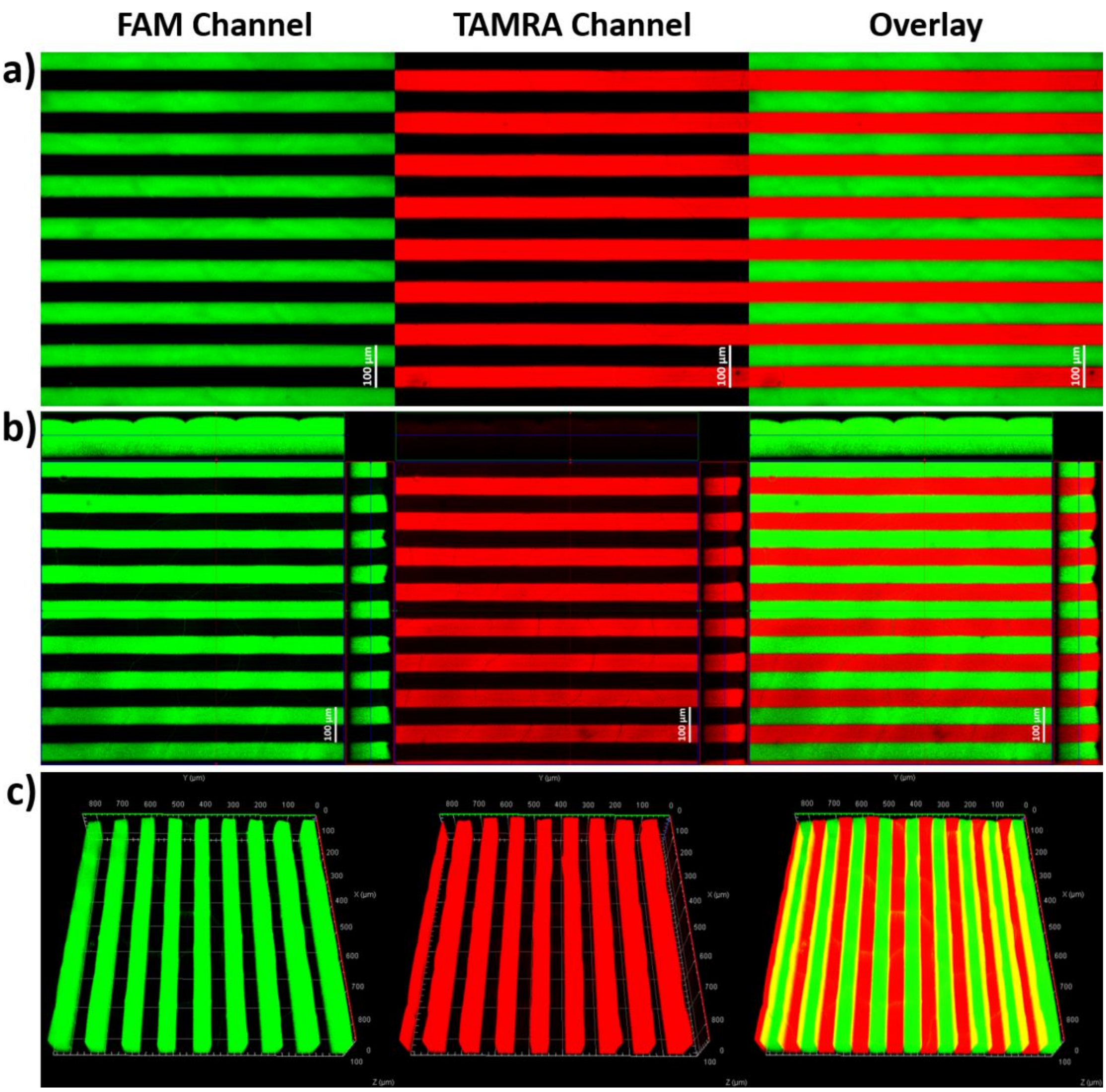


**Supplementary Figure 1.** Light-induced orthogonal patterning of a hydrogel exhibiting dual functionalities. The polyacrylamide (1)-crosslinked hydrogel is functionalized with the o-nitrobenzylphosphate ester nucleic acids (2) and (3). Exposure of the hydrogel to light yield in the illuminated domains the activated strand (3) and retain in the non-irradiated domains the free nucleic acid strand (2). The dual patterned function allows the selective association of (3')-TAMRA and (2')-FAM to the respective domains. Confocal fluorescence images **a**), orthogonal view **b**), and three-dimensional fluorescence imaging reconstruction **c**) of the dual functional DNA strand patterned hydrogel film by using stripe-like pattern containing photomask. Scale bars are 100 µm. N = 4 independent experiments were performed.

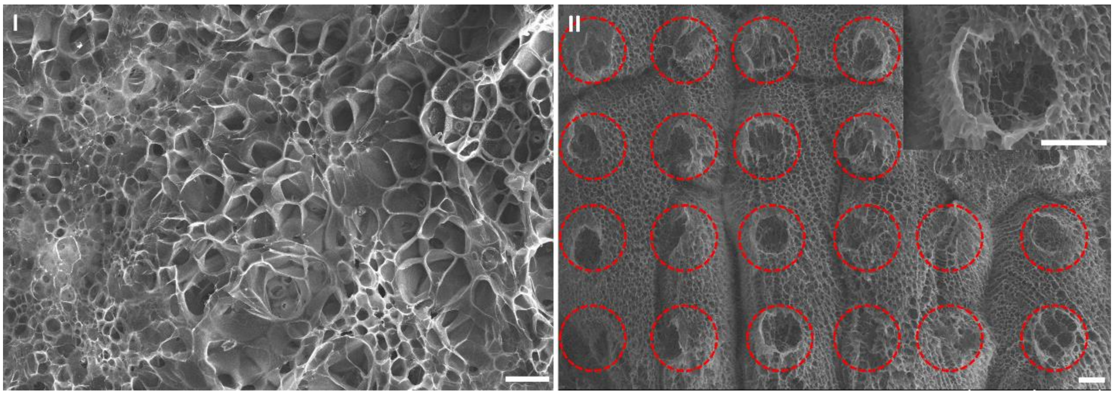


**Supplementary Figure 2.** SEM image of: Panel I– The parent hydrogel shown in Figure 5a prior to the photopatterning. Panel II– The hydrogel film after photopatterning, λ = 365 nm, according to Figure 5a. Scale bars correspond to 20 µm. N = 4 independent experiments were performed.

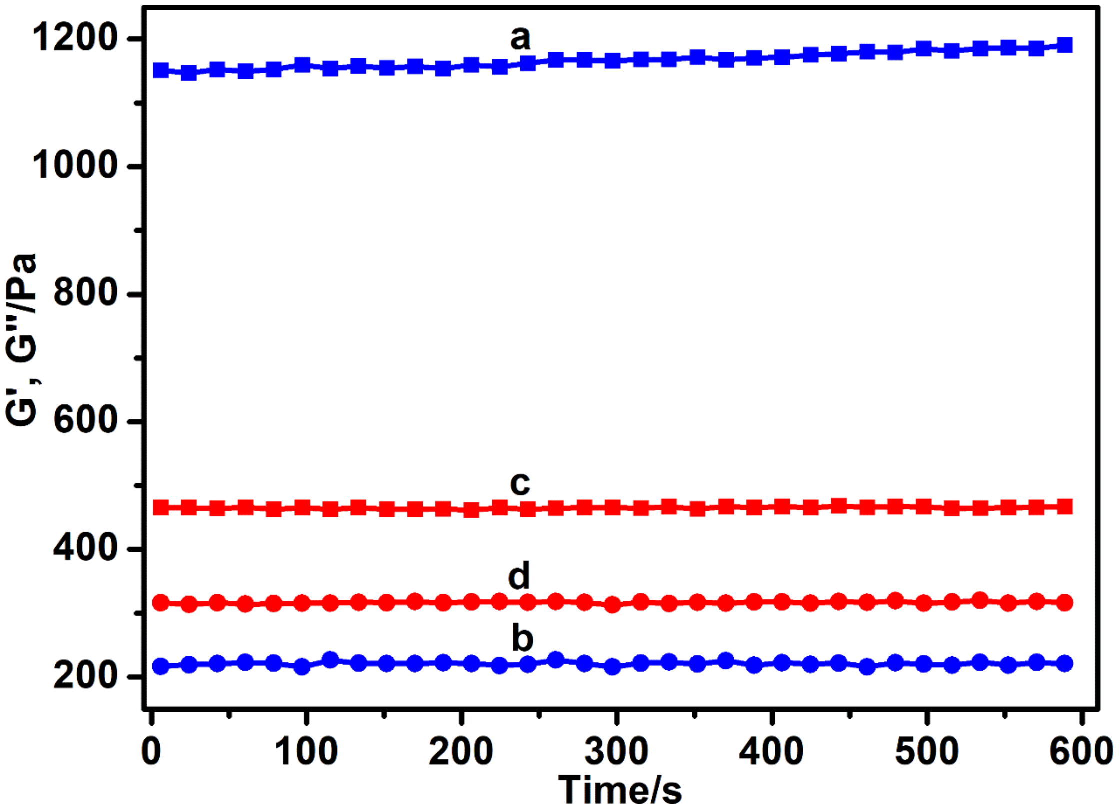


**Supplementary Figure 3.** Time-dependent sweep rheological measurements of hydrogels crosslinked with photocleavable crosslinker DNA strands (4) without UV irradiation and with 5 min UV irradiation. Curves a and b corresponding to the storage modulus G’ and loss modulus G” before UV irradiation. Curves c and d corresponding to the storage modulus G’ and loss modulus G’’ after 5 min UV irradiation.

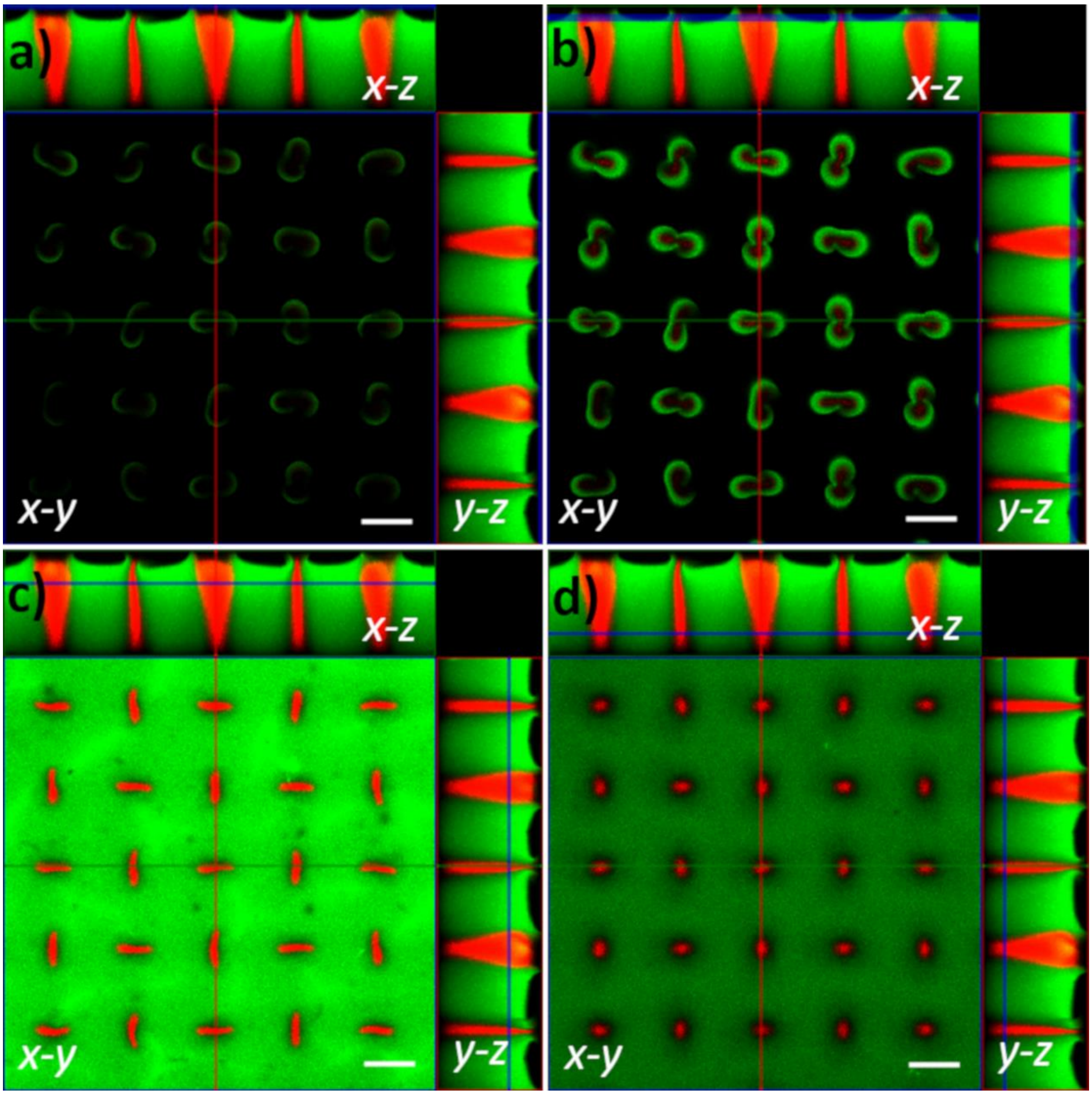


**Supplementary Figure 4.** Orthogonal views of the patterned DNA based hydrogels after DNA-directed HCR-induced programmable expansion. **a**)→**d**) show different *Z* position imaging from top surface of the hydrogel to the bottom surface of the hydrogels. Scale bars, 50 µm. N = 4 independent experiments were performed.

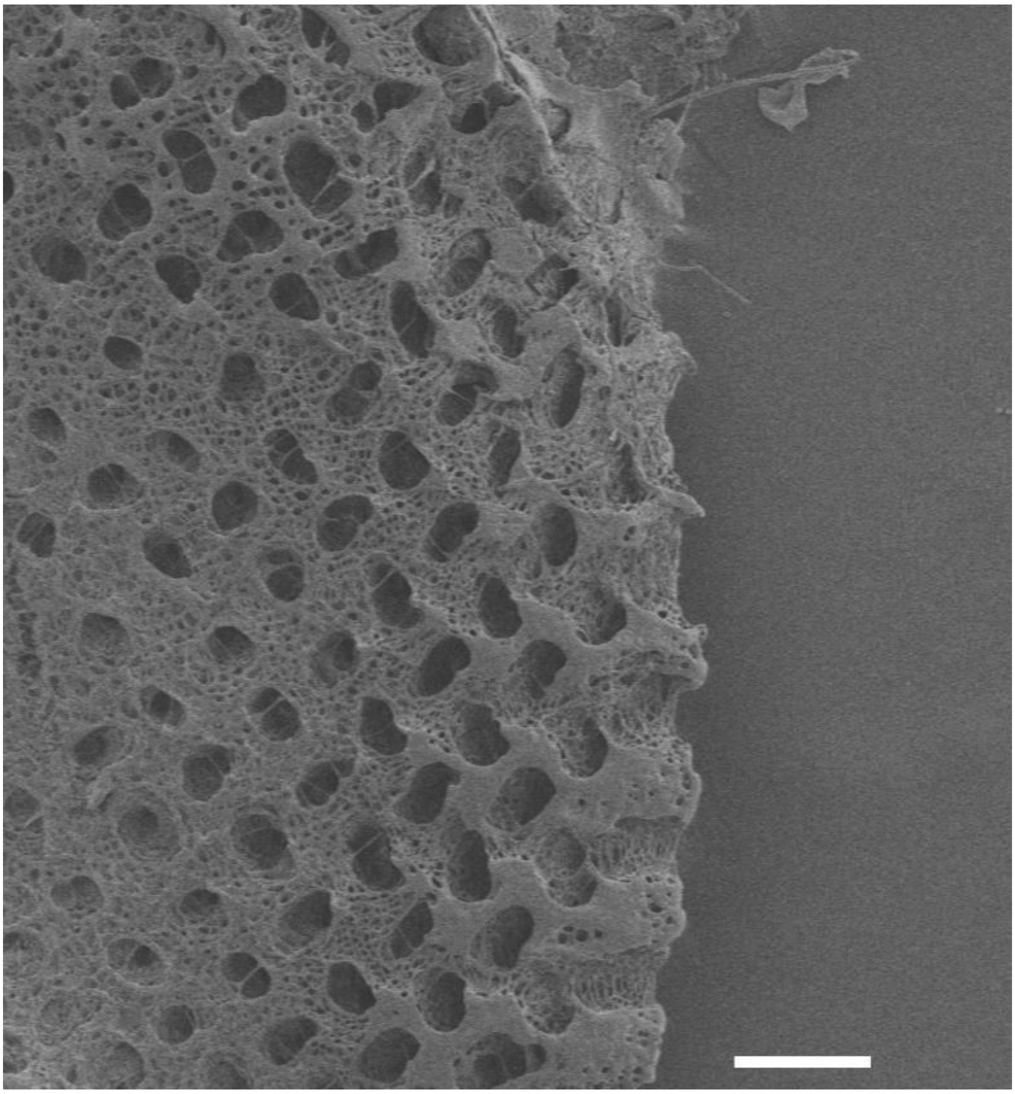

**Supplementary Figure 5.** SEM image of patterned hydrogels after HCR process with orthogonally arranged ellipsoid shapes. Scar bar is 100 nm. N = 4 independent experiments were performed.

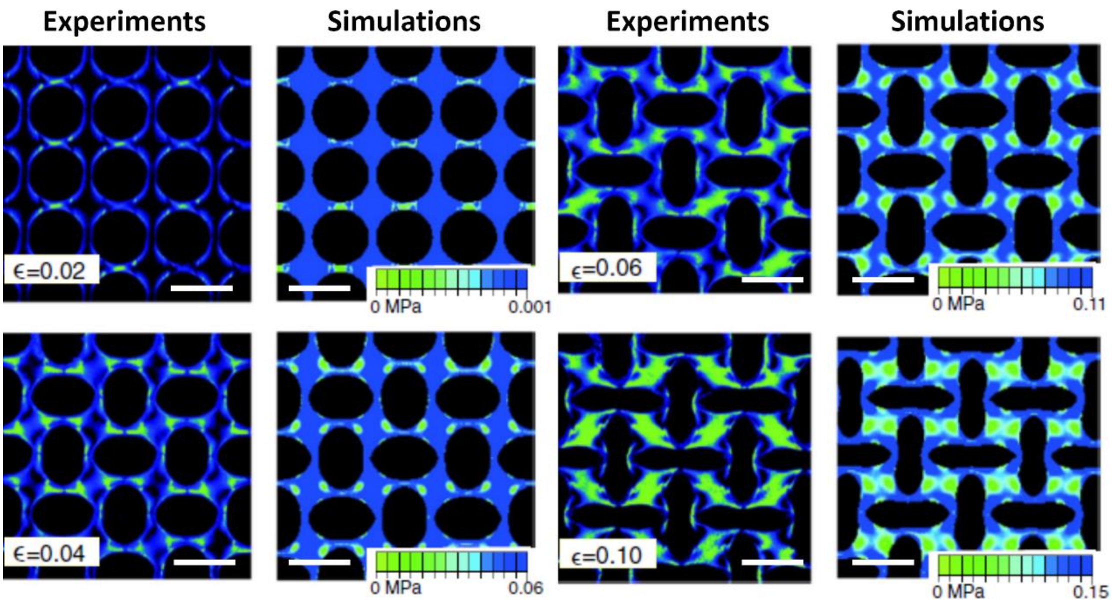


**Supplementary Figure 6.** Experimental (left) and numerical (right) images of the square lattice at different levels of macroscopic strain: 2%, 4%, 6%, and 10%. The experimental samples were viewed through cross polars and the colors give a qualitative indicator of the regions of localized stress which provide a useful comparison with the calculated principal stress levels. Scale bars correspond to 1 cm. Reproduced with permission from Ref. [1]. Copyright 2007 American Physical Society.

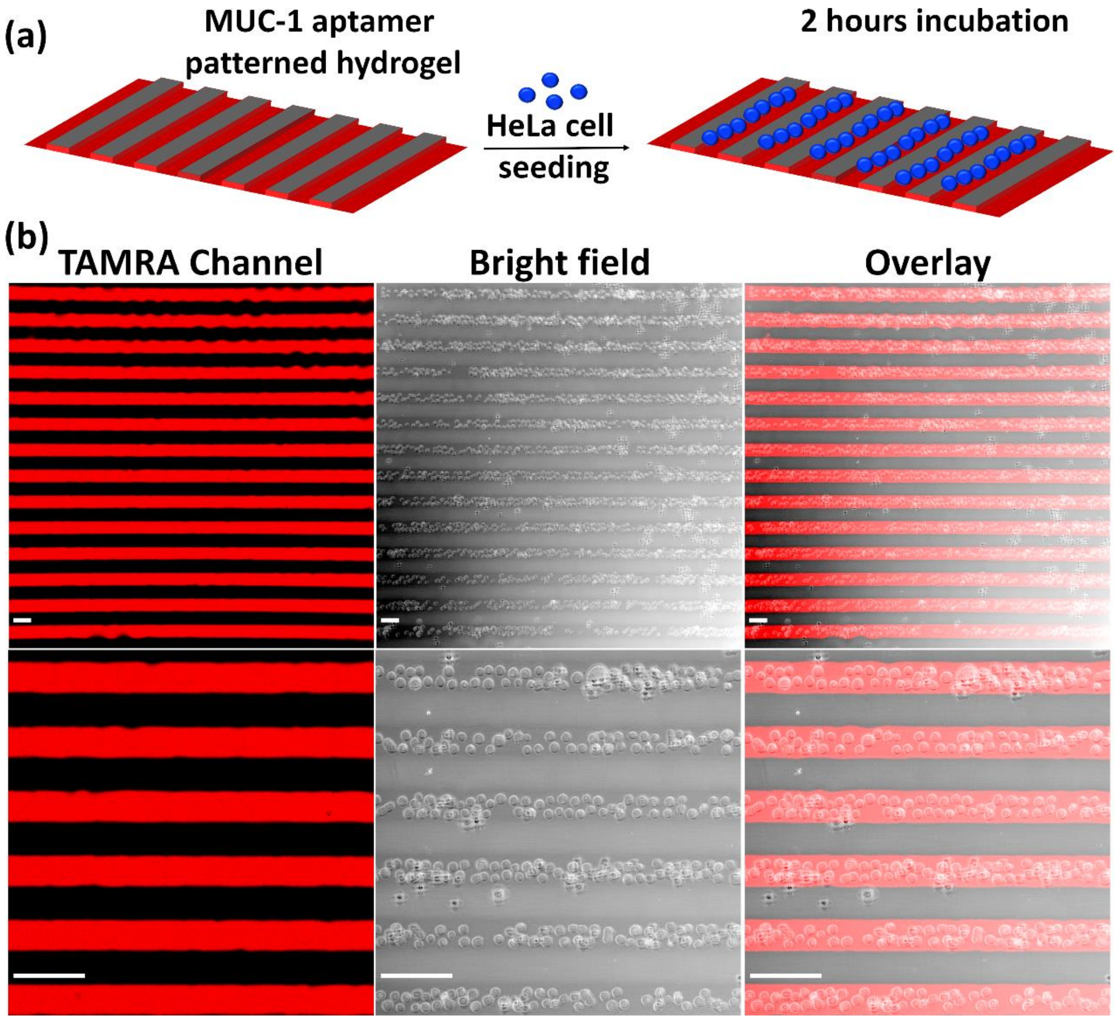


**Supplementary Figure 7.** (**a**) The patterned population-level control of HeLa cells binding in confined MUC-1 aptamer modified domains is achieved through 2D culture of cells on MUC-1 aptamer patterned hydrogel. (**b**) Fluorescent, brightfield, and overlay images highlight the cells adsorbed onto the confined MUC-1 aptamer patterned strip-like domains. N = 3 independent experiments were performed.

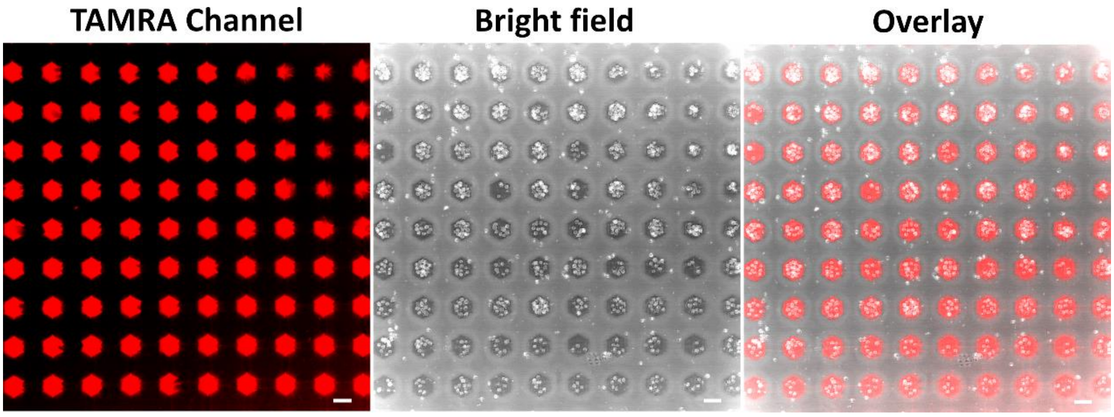


**Supplementary Figure 8.** Fluorescent, brightfield, and overlay images highlight the cells selectively adsorbed onto the confined MUC-1 aptamer patterned hexagon domains. N = 3 independent experiments were performed.

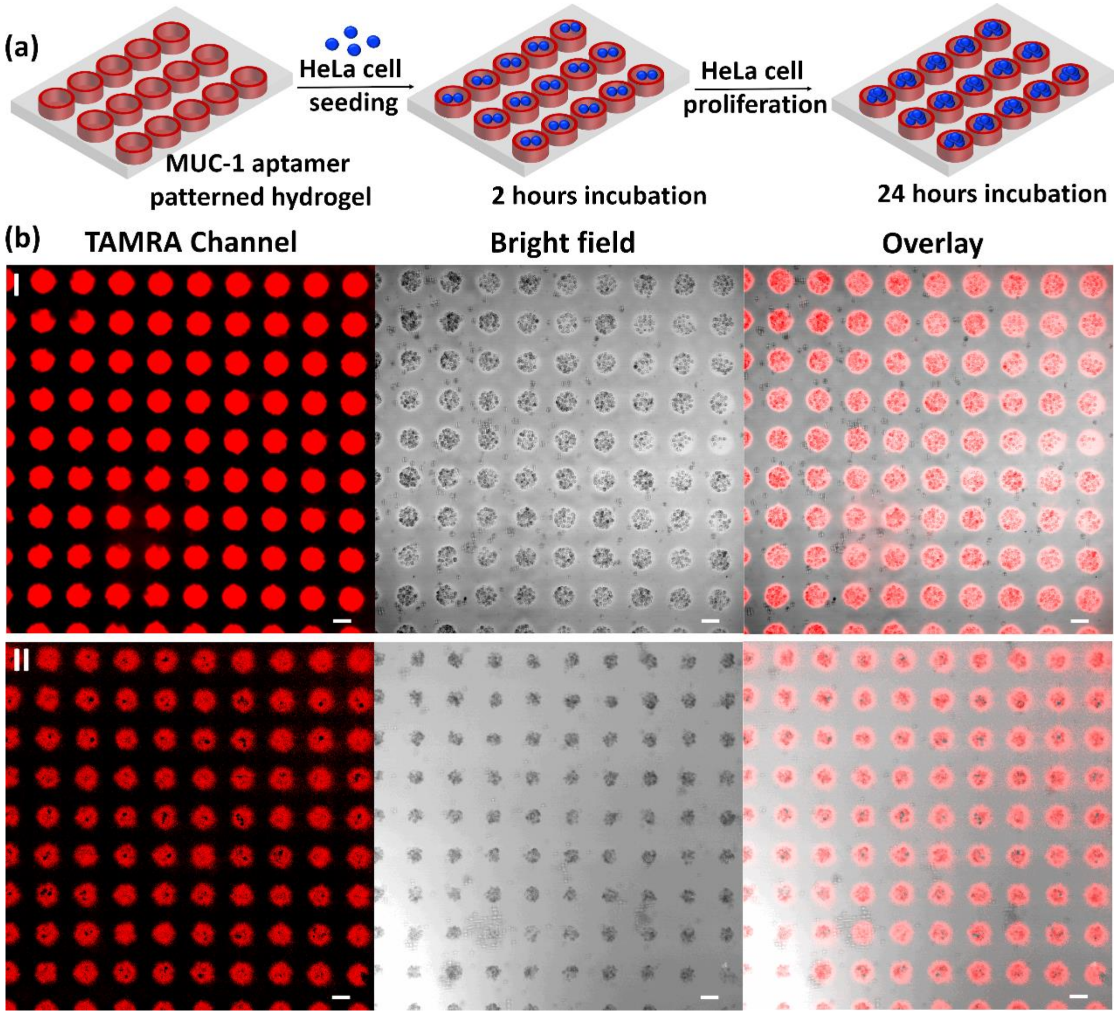


**Supplementary Figure 9.** (**a**) The patterned population-level control of HeLa cells binding and proliferation within confined MUC-1 aptamer modified domains is achieved through 2D culture of cells on MUC-1 aptamer patterned hydrogel. (**b**) Fluorescent, brightfield, and overlay images highlight the cells binding and proliferation within the confined MUC-1 aptamer patterned circular domains with 2 hours incubation (panel I) and 24 hours incubation (panel II). Scale bars are 50 µm. N = 3 independent experiments were performed.

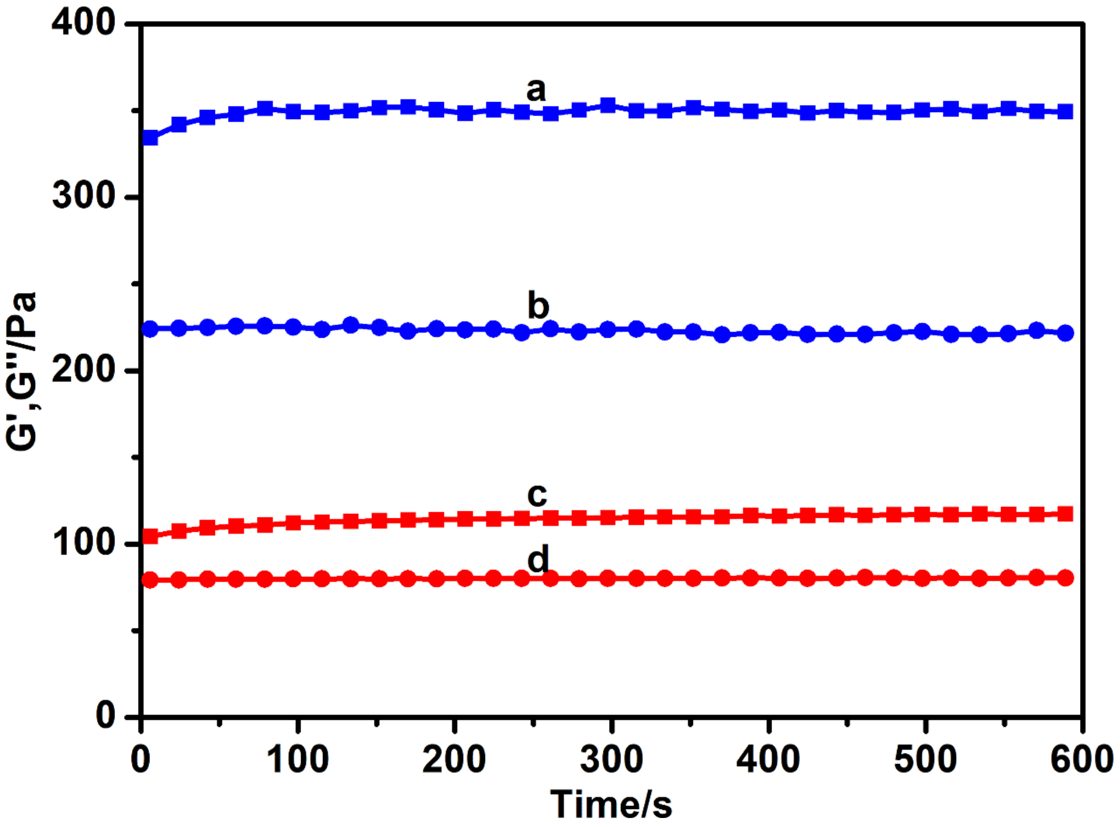


**Supplementary Figure 10.** Time-dependent sweep rheological measurements of hydrogels crosslinked with photocleavable crosslinker DNA strands (4) with 5 min UV irradiation before cell culture and after cell culture. Curves a and b corresponding to the storage modulus G' and loss modulus G" before cell culture. Curves c and d corresponding to the storage modulus G' and loss modulus G'' after 24 h cell culture.

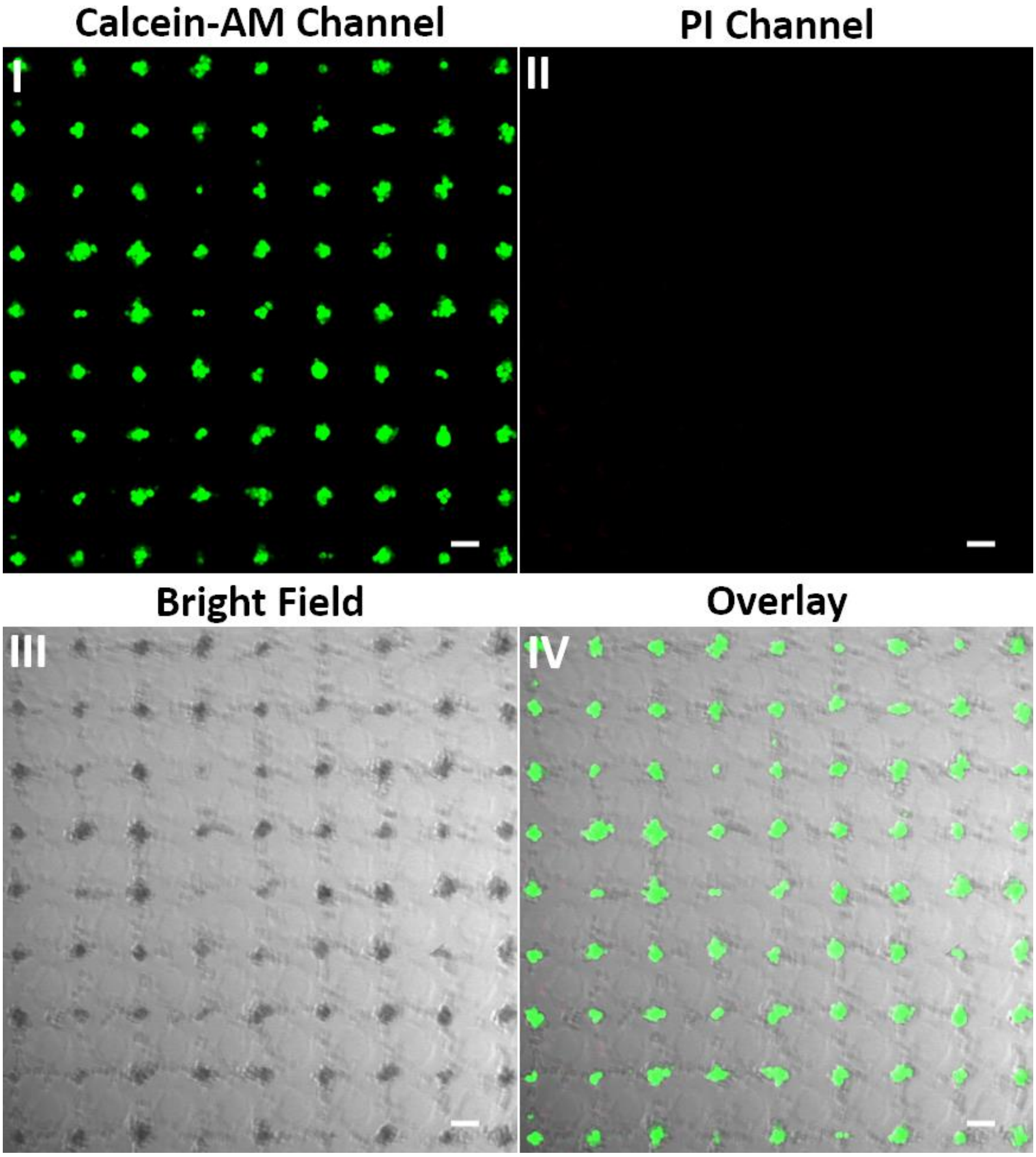


**Supplementary Figure 11.** Confocal fluorescence images of proliferated HeLa cells on patterned hydrogel surface. HeLa cells were cultured for 24 hours and then stained with calcein-AM and PI to verify the viability of the cells. Panel I– Fluorescence microcopy image (Calcein-AM channel) of the calcein-AM stained living HeLa cells proliferated in the patterned domains. Panel II– Fluorescence microcopy image (PI channel) of the PI-stained dead HeLa cells in the patterned domains. Panel III–Bright field image of the HeLa cells linked to the confined circular domains modified with MUC-1 aptamer. Panel IV– Overlay of panel I, panel II, and panel III. Scale bars are 50 μm. N = 3 independent experiments were performed.

**Reference:**

[1] Mullin, T., Deschanel, S., Bertoldi, K., Boyce, M. C. Pattern transformation triggered by deformation, Physical Review Letters, 2007, 99, 084301.
