# Supplementary material for: Spatiotemporal patterning of photoresponsive DNA-based hydrogels to tune local cell responses
Source: Nat Commun. 2021 Apr 22;12:2364. doi: 10.1038/s41467-021-22645-8 (PMC8062675; doi:10.1038/s41467-021-22645-8)
Supplement: Supplementary file 3 — Description of Additional Supplementary Files [file 41467_2021_22645_MOESM3_ESM.pdf]

Description of Additional Supplementary Files

Title: Supplementary Movie 1.

Description: Video of the computerized reconstructed 3D patterned hydrogel in Figure 2c.

Title: Supplementary Movie 2.

Description: Video corresponding to the reconstructed 3D patterned hydrogel in Figure 3c.

Title: Supplementary Movie 3.

Description: Visualization of the optical profiling of the reconstructed 3D patterned hydrogel in Figure 5c.
